# Antioxidant Activity and Protective Effects of Enzyme-Extracted *Oudemansiella radiata* Polysaccharides on Alcohol-Induced Liver Injury

**DOI:** 10.3390/molecules23020481

**Published:** 2018-02-23

**Authors:** Xiuxiu Wang, Min Liu, Chen Zhang, Shangshang Li, Qihang Yang, Jianjun Zhang, Zhiyuan Gong, Jiandong Han, Le Jia

**Affiliations:** 1 Institute of Agricultural Resources and Environment, Shandong Academy of Agricultural Science, Key Laboratory of Wastes Matrix Utilization, Ministry of Agriculture, Jinan 250100, China; xiux_wang@163.com (X.W.); sdgzy2656@126.com (Z.G.); 2 College of Life Science, Shandong Agricultural University, Taian 271018, China; lmliumin1991@sina.com.cn (M.L.); zhang.chen1@foxmail.com (C.Z.); shangshangli2012@163.com (S.L.); yangqh95@163.com (Q.Y.); zhangjj@sdau.edu.cn (J.Z.)

**Keywords:** enzyme-extracted *Oudemansiella radiata* polysaccharides, antioxidant, hepatoprotective effects, alcoholic liver diseases

## Abstract

This work was to examine the antioxidation in vitro and hepatoprotective effects of enzyme-extracted *Oudemansiella radiata* polysaccharides (En-OPS) on alcohol-induced liver damage in mice. The antioxidant activities were determined according to the scavenging effects of En-OPS on hydroxyl, superoxide, and 1,1-diphenyl-2-picrylhydrazyl (DPPH) radicals, and the level of reducing power. En-OPS showed hepatoprotective activities on decreasing the serum levels of aspertate aminotransferase (AST), alamine aminotransferase (ALT), and alkaline phosphatase (ALP), as well as hepatic lipid levels of total cholesterol (TC) and triacylglycerols (TG). En-OPS treatment reversed the acute impairment induced by alcohol consumption, including reactive oxygen species (ROS) generation, malondialdehyde (MAD), and lipid peroxide (LPO) elevation; and superoxide dismutase (SOD), GSH peroxide (GSH-Px), catalase (CAT), and total antioxidant capacity (T-AOC) impairment. The En-OPS effectively ameliorated alcohol metabolism by activating alcohol dehydrogenase (ADH) and aldehyde dehydrogenase (ALDH), and reducing cytochrome P450 2E1 (CYP2E1) levels. Furthermore, the histopathological observations also displayed that En-OPS could alleviate liver damage. These results indicated that En-OPS could be suitable to be an ingredient of preventing alcoholic liver diseases (ALD). In addition, the preliminary structure characteristics of En-OPS were also analyzed by Fourier transform infrared (FT-IR) spectroscopy and a gas chromatography-flame ionization detector (GC-FID).

## 1. Introduction

Alcohol consumption is widespread throughout the world. Moderate alcohol use may have some beneficial effects on health, such as promoting metabolism and reducing the risk of heart disease, but excessive drinking can lead to liver diseases and other health problems [1]. It has been evidenced that alcoholic liver disease (ALD) is one of the most major causes of morbidity and mortality [2]. ALD is a complex chronic process, progressing from steatosis (fatty liver) to steatohepatitis, fibrosis, and cirrhosis, and in severe cases, developing into hepatocellular carcinoma, which has become a serious and challenging health issue worldwide [3]. One species of pathologic mechanism of ALD is the production of oxidative alcohol metabolites because alcohol can induce oxidative stress, lipid peroxidation, and free radicals [4]. For enhancing hepatic antioxidant capacities and improving alcoholic liver injury, a large number of synthetic hepatoprotective medicines have been explored and used widely. However, some of these hepatoprotective medicines were limited against long-term use owing to side-effects which endanger people’s health [5]. Hence, it seems a beneficial strategy to find new safe and effective natural medicines for alcohol consumers to prevent or slow down the progression of ALD [6].

Recently, researchers have given more attention to mushrooms which are known as an important source of nutrients or physiologically beneficial and natural medicines, and have been widely used in the prevention and treatment of a variety of diseases [7]. Natural polysaccharides, widely distributed in edible mushrooms, have been found to exhibit a variety of biological activities, including antioxidant, immunomodulatory, antitumor, and other medicinal activities [8]. For instance, the polysaccharides from *Pleurotus eryngii* and *Agrocybe cylindracea* exhibited antitumor and antioxidant activities, respectively [9,10]. According to the literature, polysaccharides of some mushrooms have protective effects on acute liver toxicity in rats [11]. Thus, it seems necessary and significant to explore mushroom polysaccharides as potentially natural and effective therapeutic strategies for the treatment of ALD [12]. 

*Oudemansiella radicata*, an edible and medicinal fungi belonging to the genus *Oudemansiella* of the family Tricholomataceae, is distributed on the soil surface or rotted wood located in broad-leaved forests [13]. It is reported that its polysaccharides possess many biological activities, including immunologically-stimulating and anti-cancer properties [14]. The present literature has mainly focused on its antifungal antibiotic properties, metabolite oudenone, and the effects of lead and cadmium on the mushroom [15]. However, scarce literature about enzyme-extracted *O. radicata* polysaccharides (En-OPS) of the protective effects on acute alcohol-induced liver injury has been published. The objectives of this work were to determine the hepatoprotective effects and antioxidant activities of En-OPS on acute alcohol-induced liver injury in mice.

## 2. Results 

### 2.1. Antioxidant Capacities In Vitro

**Figure 1:**
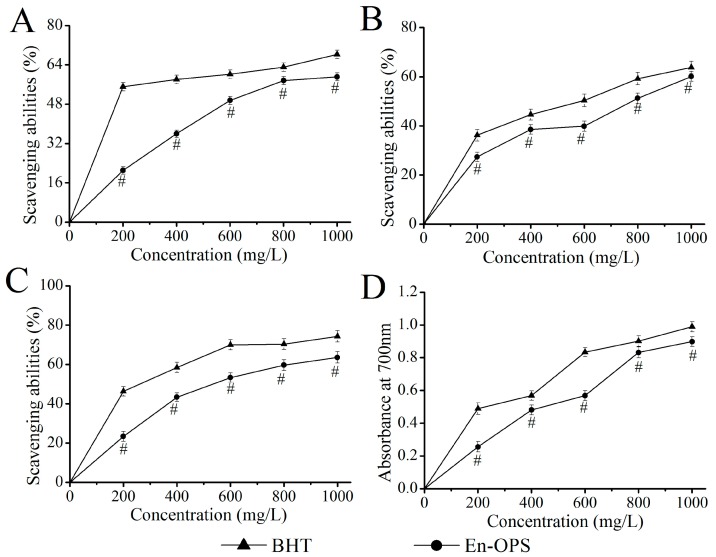
Antioxidant activities of En-OPS in vitro. (**A**) hydroxyl radical-scavenging abilities; (**B**) superoxide radical-scavenging abilities; (**C**) 1,1-diphenyl-2-picrylhydrazyl radical-scavenging abilities; and (**D**) reducing power. “#” indicated that the values differ significantly at *p* < 0.05 (En-OPS compared with BHT at different concentration). The statistical significance of the differences between the groups were analyzed using Tukey’s tests in the software package (SAS 9.3). A value of *p* < 0.05 was regarded as statistically significant.

As illustrated in Figure 1A, the scavenging effects of En-OPS and butylated hydroxytoluene (BHT) on hydroxyl radicals were correlated with concentrations. At a concentration of 1000 mg/L, the scavenging activities of En-OPS and BHT were 59.11% ± 1.64% and 68.27% ± 1.71%, demonstrating that En-OPS showed strong scavenging abilities on hydroxyl radicals. The EC_50_ values of En-OPS and BHT were 651.04 ± 2.81 and 121.49 ± 2.09 mg/L, respectively. 

As shown in Figure 1B, the scavenging effects of En-OPS and BHT on superoxide radicals displayed a dose-dependent manner at all tested concentrations. When the concentration of polysaccharide was 1000 mg/L, the scavenging abilities of En-OPS (60.09% ± 2.01%) and BHT (63.82% ± 2.49%) were similar. Moreover, EC_50_ values of En-OPS and BHT for scavenging superoxide radicals were 739.96 ± 2.87 and 498.35 ± 2.71 mg/L, respectively, indicating that En-OPS had a strong capability of scavenging superoxide radicals.

The 1,1-diphenyl-2-picrylhydrazyl radical (DPPH) scavenging activities of En-OPS and BHT were 63.59% ± 2.83% and 74.23% ± 2.89% at the dose of 1000 mg/L, respectively (Figure 1C). Furthermore, the EC_50_ values of En-OPS and BHT reached 555.69 ± 2.75 mg/L and 240.32 ± 2.38 mg/L, respectively. 

The Figure 1D showed the reducing power of En-OPS was increased from 0.26 ± 0.03 to 0.89 ± 0.03 with the increasing concentrations from 200 mg/L to 1000 mg/L, proving that En-OPS had relatively high reducing power in vitro.

### 2.2. Serum Enzyme Activities

**Figure 2:**
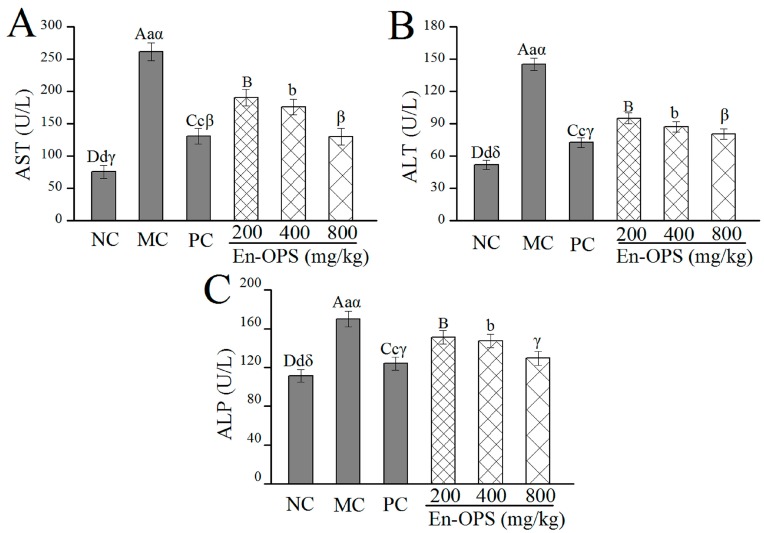
Effects of En-OPS on the enzymic activities in serum of mice. (**A**) aspertate aminotransferase; (**B**) alamine aminotransferase; and (**C**) alkaline phosphatase. The values were reported as the mean ± S.D. of 10 mice per group. Bars with different letters were significantly different (*p* < 0.05). The statistical significance of the differences between the groups were analyzed using Tukey’s tests in the software package (SAS 9.3). A value of *p* < 0.05 was regarded as statistically significant.

The effects of En-OPS on serum enzyme activities of aspertate aminotransferase (AST), alamine aminotransferase (ALT), and alkaline phosphatase (ALP) in mice were presented in Figure 2. Compared with the normal control (NC) group, the activities of AST, ALT, and ALP in the model control (MC) group were significantly elevated (*p* < 0.05), indicating that the alcohol-induced liver damage model of mice was successfully established. The AST and ALT activities reached 129.47 ± 12.99 U/L and 80.41 ± 4.89 U/L in En-OPS group at the dosage of 800 mg/kg (Figure 2A,B), which were remarkably lower than those in the MC group (261.23 ± 13.59 U/L, 145.21 ± 5.87 U/L), respectively. As for ALP, the minimum activity reached 129.54 ± 7.12 U/L, which was lower than that in the MC group (170.01 ± 7.86 U/L). Moreover, bifendatatum was also shown to decrease the serum enzyme activities in the PC group. Apparently, the AST, ALT, and ALP activities of serum in mice treated with En-OPS were reduced in a dose-dependent manner, showing the most significant hepatoprotective effect at a dose of 800 mg/kg.

### 2.3. Effect of En-OPS on Hepatic Antioxidant Activities

**Figure 3:**
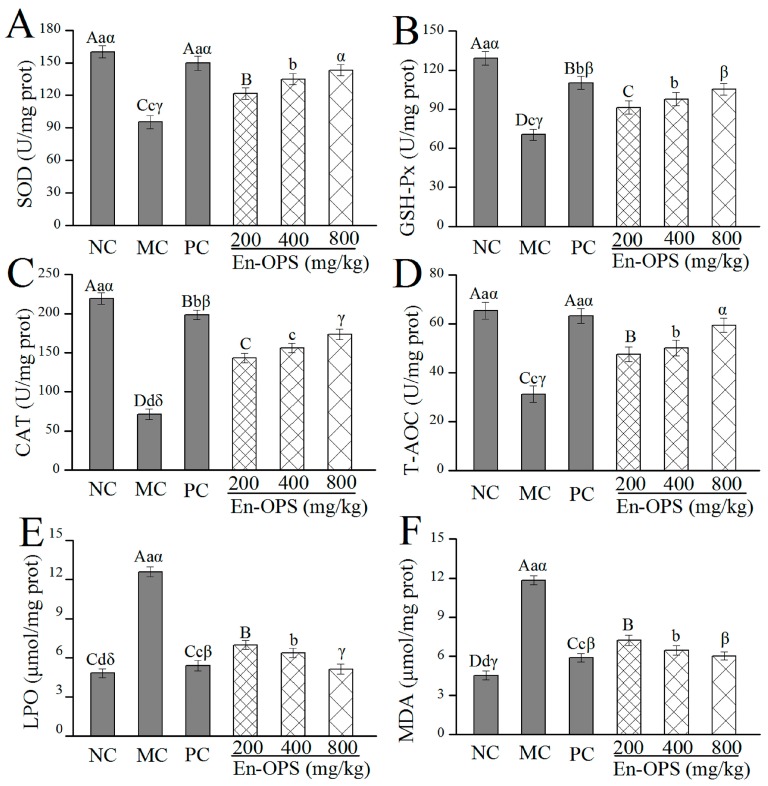
Effects of En-OPS on biochemical parameters in liver of mice. (**A**) superoxide dismutase; (**B**) GSH peroxide; (**C**) catalase; and (**D**) total antioxidant capacity, and contents of (**E**) lipid peroxide and (**F**) malondialdehyde. The values were reported as the mean ± S.D. of 10 mice per group. Bars with different letters were significantly different (*p* < 0.05). The statistical significance of the differences between the groups were analyzed using Tukey’s tests in the software package (SAS 9.3). A value of *p* < 0.05 was regarded as statistically significant.

In order to analyze the antioxidant activity of En-OPS in vivo, the six hepatic indicators including superoxide dismutase (SOD), GSH peroxide (GSH-Px), catalase (CAT), total antioxidant capacity (T-AOC), lipid peroxide (LPO), and malondialdehyde (MAD) were investigated (Figure 3). The activities of SOD, GSH-Px, CAT, and T-AOC in MC group (Figure 3A–D) were decreased compared with the NC group (*p* < 0.05). Whereas administration of En-OPS significantly enhanced the activities of SOD, GSH-Px, CAT, and T-AOC in a dose-dependent manner and the maximum reached 143.25 ± 5.01, 105.32 ± 4.53, 173.54 ± 6.41, and 59.35 ± 2.87 U/mg prot at the dose of 800 mg/kg, which were increased by 50.25, 49.73, 143.33, and 90.47% compared with those in the MC group, respectively. Meanwhile, the bifendate-treated mice also showed significant increasement of SOD, GSH-Px, CAT, and T-AOC activities compared with acute alcohol-treated mice (*p* < 0.05). As illustrated in Figure 3E,F, there was a dramatic elevation of the LPO and MAD contents in the MC group compared with those in the NC group (*p* < 0.05). However, the pretreatment of En-OPS reduced the LPO and MAD contents, especially at the dosage of 800 mg/kg, the LPO and MAD contents reached 5.12 ± 0.38 μmol/mg prot and 6.02 ± 0.29 μmol/mg prot, which were 59.33% and 49.11% lower than those in the MC group, respectively. In addition, the LPO and MAD in the positive control (PC) group treated with bifendate were decreased as compared to acute alcohol-treated mice. These results demonstrated that the En-OPS (800 mg/kg) has a similar potency to bifendate on ethanol-induced acute liver injury.

### 2.4. Effects of En-OPS on Hepatic TC, TG, ADH, ALDH, and CYP2E1

**Figure 4:**
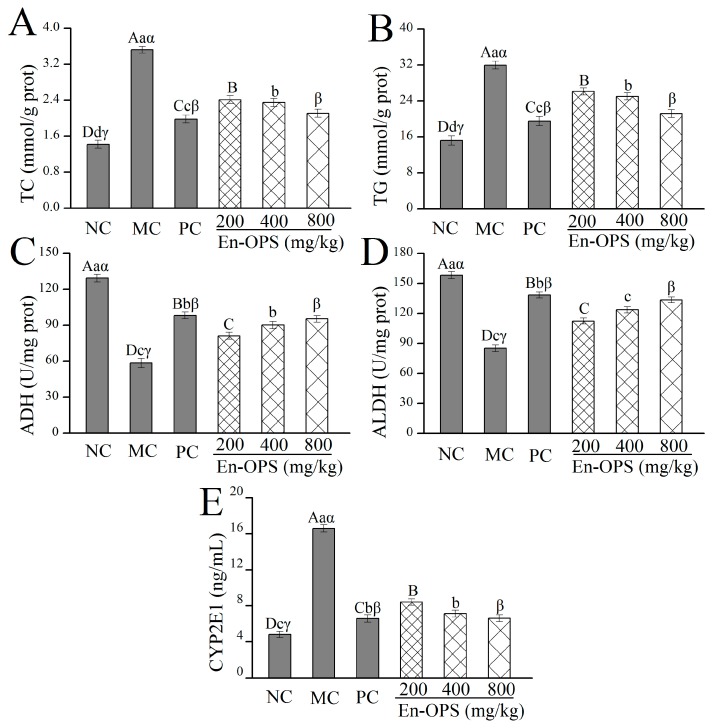
Effects of En-OPS on lipid levels and enzymic activities in liver of mice. (**A**) total cholesterol levels; (**B**) triacylglycerols levels; (**C**) alcohol dehydrogenase activities; (**D**) aldehyde dehydrogenase activities; and (**E**) cytochrome P450 2E1 levels. The values were reported as the mean ± S.D. of 10 mice per group. Bars with different letters were significantly different (*p* < 0.05). The statistical significance of the differences between the groups were analyzed using Tukey’s tests in the software package (SAS 9.3). A value of *p* < 0.05 was regarded as statistically significant.

As displayed in Figure 4A,B, there was a significantly increased level of total cholesterol (TC) and triacylglycerols (TG) in the MC group compared with the NC group (*p* < 0.05). Interestingly, the elevation of TC and TG levels could be attenuated by supplementation of En-OPS, especially at high doses. Briefly, En-OPS at the dose of 800 mg/kg suppressed the hepatic levels of TC and TG (2.11 ± 0.09 mmol/mg prot and 21.19 ± 0.85 mmol/mg prot), 40.06% and 33.68% lower than those in MC group, respectively. Simultaneously, significantly lower levels of TC and TG were observed in the livers of mice treated with bifendate (*p* < 0.05). These data demonstrated that En-OPS has superior effects on lowering the hepatic lipid levels.

**Figure 5:**
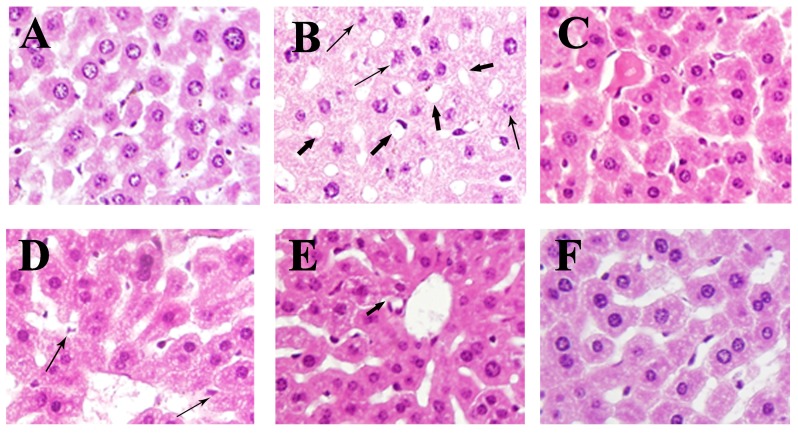
Effects of En-OPS on hepatic cells in liver tissue of alcohol-intoxicated mice. (**A**) normal control groups; (**B**) model control groups; (**C**) positive control groups; and (**D**–**F**) groups treated with 200, 400, and 800 mg/kg En-OPS (magnification 400×). Cellular degeneration (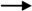), lipid droplet accumulation (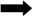).

The effects of En-OPS on hepatic activities of alcohol dehydrogenase (ADH) and aldehyde dehydrogenase (ALDH) were assessed (Figure 4C,D), the mice in MC group showed significant decline in the activities of ADH and ALDH as compared with NC group (*p* < 0.05), suggesting that alcohol metabolism in the alcohol dehydrogenase oxidation system was partially inhibited. Moreover, at the dosage of 800 mg/kg treated with En-OPS, the ADH and ALDH activities were 95.32 ± 2.75 U/mg prot and 133.56 ± 2.87 U/mg prot, which were 62.99% and 56.91% higher than those in MC group. Additionally, there was a significant reduction of cytochrome P450 2E1 (CYP2E1) level in MC group compared with the NC group (*p* < 0.05) (Figure 5E). The CYP2E1 level of En-OPS at 800 mg/kg was 6.61 ± 0.38 ng/mL, 60.15% lower than that in MC group. Interestingly, the PC group also manifested significant elevation of ADH and ALDH activities, and significant reduction of CYP2E1 level as compared with the MC group (*p* < 0.05). The present results indicated that En-OPS has potential effects for remitting alcohol-induced liver damage.

### 2.5. Effects of En-OPS on Liver Histopathology 

Normal liver cells structure with aligned nuclei, unspoiled cytoplasm, and visible central veins were observed in liver tissue of NC group (Figure 5A). In contrast, the liver tissue of mice in MC group exhibited diffuse cellular degeneration and hepatic lipid droplet accumulation were observed in liver tissue of MC group (Figure 5B), indicating that the liver cells were seriously damaged. Bifendate administration (Figure 5C) can alleviate histopathological status in the MC group. Moreover, En-OPS pretreatment improved the alcohol-induced abnormalities effectively in the architecture of liver tissue in a dose-dependent manner from 200 mg/kg to 800 mg/kg (Figure 5D–F). These findings declared that En-OPS has potential effects on the protection of mice livers against alcohol-induced liver damage.

### 2.6. FT-IR Spectrum Analysis

**Figure 6:**
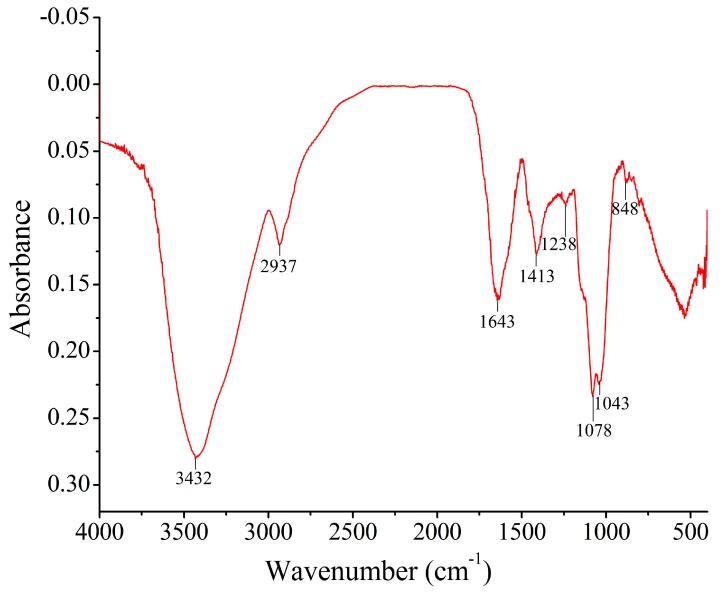
Fourier transform infrared (FT-IR) spectrum of En-OPS.

A typical Fourier transform infrared (FT-IR) spectrum of En-OPS was depicted in Figure 6. The broad stretching peak at 3405 cm^−1^ was characteristic of hydroxyl groups, and the weak absorption peaks at 2927 cm^−1^ were ascribed to the C–H stretching vibration, which were characteristic absorption peaks of polysaccharide. Moreover, the band at 1640 cm^−1^ was due to the stretching vibration of C=O and the band in the region of 1420 cm^−1^ was likely related to O–H bond bending vibrations. The absorption peaks at 1200–1000 cm^−1^ were ascribed to sugar ring vibrations overlapping with stretching vibrations of C–OH and the C=O=C. The band at about 840 cm^−1^ indicated the existing of α-glycosidic. Hence, the En-OPS had the typical absorption of polysaccharides [16].

### 2.7. Monosaccharide Compositions Analysis

**Figure 7:**
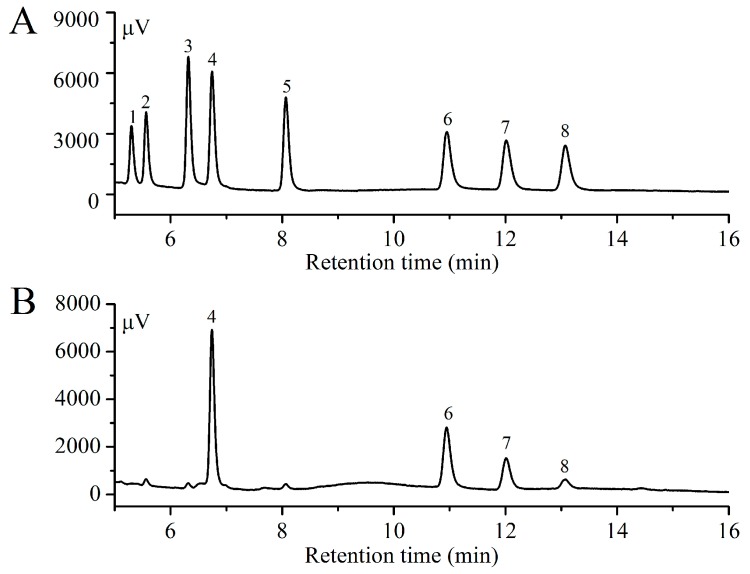
Gas chromatography (GC) chromatograms of monosaccharide. (**A**) Standard sugars; (**B**) En-OPS. Peaks: (1) rhamnose, (2) fucose, (3) ribose, (4) arabinose, (5) xylose, (6) mannose, (7) galactose, and (8) glucose.

The monosaccharide composition of En-OPS was identified by comparing the retention times with standard samples (Figure 7A). As presented in Figure 7B, En-OPS was mainly composed of four different monosaccharides—including arabinose, mannose, galactose, and glucose—with mass percentages of 35.58, 26.03, 17.45, and 20.94%, respectively, and the molar ratio was 1.64:1.49:1:1.2.

## 3. Discussion

Oxidative stress is thought to play a major role in the pathogenesis of alcohol-induced liver injury, which induces the damage of tissue by the imbalance of pro-oxidants and antioxidants. Alcohol mediates oxidative stress in a variety of ways involving the generation of reactive oxygen species (ROS), lipid peroxidation, and depletion of endogenous antioxidant functions [17]. ROS is generated by normal metabolism or exogenous factors, including a series of free radicals, such as hydroxyl, hydrogen peroxide, and superoxide radicals. Whereas acute alcohol consumption can result in the excess ROS, it may cause toxicity, DNA instability, impaired membrane integrity declined enzymatic activities, and further trigger many diseases; such as cancer, rheumatoid arthritis, liver injury, atherosclerosis, inflammation, and aging [18,19]. Polysaccharide, a natural antioxidant, contributed to removing excess ROS and maintain the delicate balance of biological systems [20]. In the present work, the in vitro antioxidant activities of En-OPS were measured by evaluating the scavenging effects on hydroxyl, superoxide, and DPPH radicals, and the level of reducing power. The hydroxyl radical, as reported in the literature was attributed to be the most reactive radical and induced severe damage of tissues and cells as an active initiator for lipid peroxidation [21]. Simultaneously, the scavenging capacity of polysaccharides towards hydroxyl radicals was directly related to its antioxidant activity [22]. Although superoxide radicals are a relatively weak oxidant compared to other free radicals and oxidizing agents, it is a precursor of singlet oxygen and hydroxyl radicals that indirectly activates lipid peroxidation [23]. Additionally, the DPPH free radical is a stable free radical and widely used to estimate the scavenging abilities of natural compounds, and the possible mechanism may be that natural compounds can transfer either an electron or a hydrogen atom to DPPH [24]. Furthermore, numerous studies have demonstrated that antioxidant activity have a direct, positive correlation with the reducing power [25]. In the present work, En-OPS displayed remarkable reducing power and scavenging effects on hydroxyl, superoxide, and DPPH radicals. Compared with the polysaccharides from *Agaricus bisporus* and polysaccharides from *Pleurotus ostreatus*, the En-OPS showed superior radical scavenging abilities and reducing power at the same tested concentrations [26,27]. In addition, it is known that cells and tissues protect themselves against oxidative damage by scavenging ROS and the terminating chain reaction of free radicals in vivo by using enzymatic and non-enzymatic antioxidant defense system. The antioxidant enzymes—such as SOD, GSH-Px, and CAT—are regarded as the primary antioxidant defense mechanisms against oxidative stress. In brief, SOD first reduces the superoxide radical to H_2_O_2_ and O_2_, and GSH-Px and CAT catalyze H_2_O_2_ to H_2_O and O_2_, thereby preventing hydroxyl radical formation. The non-enzymatic antioxidant capacity on organs was determined by analyzing T-AOC values [16,28]. In biological systems, MDA is considered as the toxic aldehydes produced by lipid peroxidation, and LPO is the product of oxygen free radicals and polyunsaturated fatty acid reactions. Commonly, the formation of MDA and LPO in liver is considered a biomarker of cell membrane disruption and cell damage [29,30]. Results of this paper showed that the significant decrease of SOD, GSH-Px, CAT, and T-AOC activities, as well as remarkable increases of MDA and LPO contents in alcohol-intoxication mice, indicated the raised liver oxidative damage. Conversely, the En-OPS pretreatment dramatically stimulated the activities of SOD, GSH-Px, CAT, and T-AOC and significantly reduced the contents of MDA and LPO in the liver. In conclusion, En-OPS showed potential protective effects against acute alcohol-induced liver injury, which might be associated with the scavenging effects on free radicals and the inhibition of lipid peroxidation. These results agree well with the previous findings by Cui et al. of *Aloe vera* polysaccharides [31], and Xiao et al. of *Lycium barbarum* polysaccharides [32].

The increased AST, ALT, and ALP activities in serum are usually considered hallmarks of hepatic damage [33]. Furthermore, more and more evidence indicates that acute alcohol consumption impairs the operations of fatty acid metabolism through reducing the AMP-activated protein kinase activity in liver [34]. The hepatic TC and TG levels are a common response to fat accumulation in the liver, which, in turn, leads to the decrease of liver function [35]. In the current work, the significant increase of AST, ALT, and ALP activities in serum, and TC and TG levels in the liver, were produced in alcohol-induced mice as compared with that in the NC group. As we expected, En-OPS has a potential protective effect on ALD by preventing the accumulation of these enzymes and substances. Moreover, similar results were also confirmed by Zhang et al. for *Lepidium meyenii* polysaccharides [36].

Alcohol metabolism is usually considered to be one of the major causes of alcohol-induced liver injury [37]. The liver is a vital organ of alcohol metabolism in the body, and more than 85% of ingested ethanol is metabolized in the liver [38]. The primary alcohol metabolic enzymes included cytosolic ADH, mitochondrial ALDH, and cytochrome P450 2E1 (CYP2E1). The alcohol was decomposed into acetaldehyde by ADH and, subsequently, into acetate by ALDH, and then into carbon dioxide and water by the tricarboxylic acid cycle. Thus, the severely ethanol-induced liver injury might be associated with the activities of ADH and ALDH [39]. The CYP2E1 is one of important metabolic enzymes, which catalyzes the conversion of ethanol to acetaldehyde but, at the same time, also produces high amounts of ROS. Therefore, it is particularly correlated with the development of liver damage caused by the generation of alcohol-induced ROS [40]. The current research results show the hepatoprotective effect on polysaccharides against alcohol-induced liver injury by attenuating the expression of CYP2E1 and by enhancing the protective role of anti-oxidative defense system. Similar studies have previously shown that a wide range of natural products and their active substances have strong CYP2E1 inhibitory capacity [41,42]. In this experiment, En-OPS can increase the activities of ADH and ALDH and reduce CYP2E1 levels, indicating that En-OPS has a positive effect on alcohol metabolism.

More and more evidence indicates that enzyme-assisted extraction of polysaccharides has many characteristics—such as simplified manipulation, lower energy requirements, lower investment costs, and reduced relative viscosity—and changes the monosaccharide composition [14,43]. Additionally, it is significant to understand the component monosaccharide profiles of the polysaccharides, which contribute to the biological effects [44]. Herein, the polysaccharides by enzyme-assisted extraction contained high contents of arabinose and mannose, indicating that the two monosaccharides may play vital roles in maintaining biological functions. Similar results were also reported by Lu et al. [45]. It seemed that the chemical and structural properties of En-OPS might contribute to the hepatoprotective effects. 

## 4. Materials and Methods

### 4.1. Material and Chemicals

The fruiting body of *O. radicata* used in this experiment was obtained from Beijing Academy of Agriculture and Forestry Sciences (Beijing, China). The standard monosaccharide samples were from Sigma Chemicals Company (St. Louis, MO, USA). Diagnostic reagent kits including TC, TG, ADH, ALDH, CYP2E1, and the detection of antioxidant indices in vivo were purchased from Nanjing Jiancheng Bioengineering Institute (Nanjing, China). All the other chemicals and reagents used in present work were provided by local chemical suppliers in China. 

### 4.2. Extraction of Polysaccharide

The En-OPS were prepared according to previously reported methods [46] with minor modifications. The fruiting body was dried at 60 °C, crushed into powder using a disintegrator (Shanghai, China) and stored in dry and dark. The powder was extracted with salinase solution (2%, *w*/*v*, pH 6) at 37 °C for 4 h. The supernatant was collected by centrifugation (3600 r/min, 15 min), and mixed with three volumes of ethanol (95%, *v*/*v*). After incubation at 4 °C for 24 h, the precipitate was gathered after centrifugation (3600 r/min, 15 min). The precipitate was dissolved in distilled water, then the Sevag reagent (1-butanol/chloroform 1:5, *v*/*v*) was added. The mixed solution was placed in table concentrator and shaken for 30 min. After being static 30 min, supernatants were collected. The operation was repeated until precipitate-free [47,48]. Subsequently, the deproteinized supernatant was precipitated at ethanol concentration of 95%. After centrifugation (3600 r/min, 15 min), the precipitate was washed with anhydrous ethanol, acetone, and ether in turn to remove pigment, amino acids, and other impurities, and then lyophilized and collected. The sample solution (1 mg/mL) was prepared for UV spectroscopy analysis using a microplate spectrophotometer (Chantilly, VA, USA) and the UV spectra of En-OPS showed no absorption at 280 and 260 nm for proteins and nucleic acids, indicating that the En-OPS was homogeneous. 

### 4.3. Antioxidant Effects In Vitro

Hydroxyl radical scavenging ability was measured according to the previously reported method [49] with modification. The reaction mixture (9 mL) containing 1 mL phenanthroline (7.5 mmol/L), 1 mL ferrous sulfate (0.75 mmol/L), 5 mL phosphate buffer (0.2 mol/L, pH 7.4), 1 mL H_2_O_2_ (0.3%), and 1 mL sample (200–1000 mg/L) was incubated at 37 °C for 1 h and then the absorbance at 510 nm was measured with water as a blank and BHT as a positive control. The scavenging abilities were calculated as

Scavenging abilities of hydroxyl radical (%) = (A_blank_ − A_sample_)/A_blank_ × 100 (1)

The scavenging effect on superoxide radical was processed using the reaction mixtures containning 1 mL sample (200–1000 mg/L) and 2 mL Tris-HCl buffer (50 mmol/L, pH 8.2) at 25 °C for 20 min. After adding 0.4 mL of 1,2,3-phentriol (5 mmol/L) to terminate the reaction, the absorbance at 325 nm was measured with water as a blank and BHT as a positive control. The scavenging activities were evaluated using the equation

Scavenging abilities of superoxide radical (%) = (A_blank_ − A_sample_)/A_blank_ × 100 (2)

Scavenging activity of En-OPS toward DPPH was measured according to the reported method [50] with minor modifications. Briefly, 2 mL of various samples concentration (200–1000 mg/L) were added to 2 mL of anhydrous ethanol solution of DPPH (0.2 mmol/L, appending emulsifier) and the mixture was shaken thoroughly. After incubation in the dark for 30 min, the absorbance was determined at 517 nm with water added to 2 mL of anhydrous ethanol solution of DPPH as the blank and BHT as a positive control. The scavenging abilities were calculated as

Scavenging abilities of DPPH radical (%) = (A_blank_ − A_sample_)/A_blank_ × 100 (3)

Reducing power was assayed with minor modification of the published method [51]. One milliliter of sample (200–1000 mg/L) was mixed with 2.5 mL phosphate buffer (0.2 mol/L, pH 6.6) and 2.5 mL potassium ferricyanide (1%, *w*/*v*), and incubated in water bath at 50 °C for 20 min. After cooling with water 2.5 mL of 10% trichloroacetic acid was added and then centrifuged (3000 r/min, 10 min), and the supernatant (2.5 mL) was mixed with 2.5 mL distilled water and 2.5 mL ferric chloride (0.1%, *w*/*v*). The absorbance was measured at 700 nm using water as a blank control and BHT as a comparison.

### 4.4. Animal Experiments

A group of 60 male Kunming strain mice weighing 20 ± 2 g were purchased from Taibang Biological Products Ltd. Company (Taian, China), and the animals were maintained at animal room conditions with controlled temperature (24 ± 1 °C), humidity (55 ± 5%) and a 12 h light/dark cycle, all the mice had access to a standard laboratory pellet diet and water ad libitum. All the animals were allowed to acclimatize for one week before the experiment. The animal experimental procedures complied with the institutional animal care and use committee of Shandong Agricultural University and were conducted in accordance with the Animals (Scientific Procedures) Act, 1986 (amended 2013).

After domestication, all mice were randomly distributed into six groups of 10 mice in each group. These groups were the NC group, MC group, PC group, En-OPS low dose group, En-OPS medium dose group, and the En-OPS high dose group. In the NC and MC groups, the mice were orally administered normal saline as a blank. In the PC group, the mice were treated with bifendate (150 mg/kg). In the three dose groups, the mice were treated with a low-dose (200 mg/kg), medium-dose (400 mg/kg), and high-dose (800 mg/kg) of polysaccharide samples [52]. Gavage was processed with a syringe once daily and the entire experiment procedure lasted for 20 consecutive days. On the 21st day, all mice, except those in the NC group, were orally administered with 8 mL/kg of 50% alcohol solution every 6 h for four times to induce hepatic injury by the previous reports [42,53]. 

### 4.5. Biochemical Assays

After the final intragastrical administration, all of the mice were sacrificed under ether narcotization after 4 h of fasting and blood samples were collected without anticoagulant from eyeballs. Serum was separated from blood samples by centrifugation at 4000 r/min for 10 min. The activities of AST, ALT, and ALP of the serum were measured using an automatic biochemical analyzer (ACE, Armonk, NY, USA). Livers were excised, weighed, and homogenized immediately (1:9, *w*/*v*) in phosphate buffer (0.2 mol/L, pH 7.4 at 4 °C). The supernatant was obtained after centrifugation at 5000 r/min for 20 min at 4 °C. The hepatic activities of SOD, GSH-Px, CAT, T-AOC, ADH, and ALDH, the contents of LPO and MDA, as well as the hepatic levels of TC, TG, and CYP2E1 were measured using commercial reagent kits following the manufacturers’ instructions.

### 4.6. Histological Assays

Liver tissues of mice from different group were immersed in the 4% neutral buffered formaldehyde solution, processed and embedded in paraffin. About 5 μm thick slices were produced, stained with hematoxylin-eosin (H&E), and sequentially observed under a microscope (400× magnification) for histological analyses.

### 4.7. FT-IR Spectrum Analysis

The FT-IR spectrum was recorded using an infrared spectrometer (Nicolet 6700, Thermo Fisher Scientific, Waltham, MA, USA). Two milligrams of En-OPS were grinded with KBr powder and then pressed into pellets for FT-IR measurement in the range of 4000–400 cm^−1^.

### 4.8. Monosaccharide Composition Analysis

The monosaccharide constituents of En-OPS were determined by GC according to the described method [54]. GC was performed on Shimadzu GC-2010 instrument (Tokyo, Japan) equipped with a flame ionization detector. The dried polysaccharides (0.1 g) were hydrolyzed with 1.8 mL trifluoroacetic acid (TFA, 2 mol/L) at 110 °C for 4 h, mixed with 0.6 mL ammonium hydroxide (12 mol/L), and 0.4 mL sodium borohydride (2%, *w*/*v*). The hydrolyzed product was acetylated with 0.6 mL methylimidazole and acetic anhydride. After centrifugation, the supernate (1 μL) was injected into a capillary column of Rtx-1 (30 mm × 0.25 mm × 0.25 µm). Monosaccharide compositions were confirmed by comparison with standard monosaccharides including rhamnose, fucose, ribose, arabinose, xylose, mannose, galactose, and glucose. The relative molar ratios were analyzed by the area normalization method according to the chromatogram.

### 4.9. Statistical Analysis

All results were presented as the mean ± S.D. (standard deviation). The statistical significance of the differences between the groups were analyzed using Tukey’s tests in the SAS software package (SAS, 9.3, Inc., Cary, NC, USA). A value of *p* < 0.05 was regarded as statistically significant. 

## 5. Conclusions

The current study demonstrated that the administration of En-OPS significantly decreased serum AST, ALT, and ALP levels; inhibited MDA and LPO formation; enhanced antioxidant enzyme activities; and scavenged hydroxyl, superoxide, and DPPH radicals, indicating that En-OPS possessed potential antioxidant abilities and protective effects on acute alcohol-induced ALD. Furthermore, the characterizations showed that En-OPS was typical absorption of polysaccharides composed of arabinose, mannose, galactose, and glucose with different molar ratios. The present results suggested that the En-OPS could be used as a potentially natural and functional foods or a novel hepatoprotective agent for treatment of ALD.
